# Exploring variability of teaching & supervision at clinical clerkship teaching sites

**DOI:** 10.12669/pjms.342.14656

**Published:** 2018

**Authors:** Naghma Naeem, Margaret Elzubeir, Mohammad Al-Houqani, Luai Awad Ahmed

**Affiliations:** 1 Dr. Naghma Naeem, PhD. Department of Medical Education, College of Medicine & Health Sciences, United Arab Emirates University, United Arab Emirates; 2 Prof. Margaret Elzubeir, PhD. Department of Medical Education, College of Medicine & Health Sciences, United Arab Emirates University, United Arab Emirates.; 3 Dr. Mohammad Al-Houqani, FCCP. Department of Medical Education, College of Medicine & Health Sciences, United Arab Emirates University, United Arab Emirates; 4 Dr. Luai Awad Ahmed, PhD. Institute of Public Health, College of Medicine & Health Sciences, United Arab Emirates University, United Arab Emirates

**Keywords:** Education, Medical student, Teaching, Supervision, Variation

## Abstract

**Objective:**

To explore undergraduate medical students’ perception of variation in teaching and supervision at different clinical teaching sites.

**Methods:**

This descriptive cross-sectional study was conducted at the College of Medicine & Health Sciences, United Arab Emirates University, UAE during 2017. Four clinical teaching sites affiliated with CMHS were evaluated namely Shaikh Khalifa Medical City (SKMC), Ambulatory Care Clinics (AC), Tawam Hospital (TH) and Al-Ain Hospital (AH). An online questionnaire was administered to year five and six students.

**Results:**

The response rate was 84.4%. Overall perception of the students about their clinical clerkship experience was positive. SKMC was rated as the best teaching site with mean rating of 3.79±0.97-4.79±0.43. The highest rated item was clinical teacher’s promotion of critical thinking in students while the lowest rated item was the opportunity to take responsibility for patient care. Ambulatory Care site had a mean rating of 2.33±1.23-4.13±1.19. The highest rated item at this site was the clinical teacher encouraging students to ask questions and participate actively. At Tawam Hospital, the mean ratings ranged between 2.65±1.64-4.31±0.86 with highest rated item being ability of the students to see cases with positive clinical findings. At the Al-Ain Hospital, the mean rating was in the range of 2.79±1.45-3.81±1.11. The item rated highest here was the ability of students to see cases with positive clinical findings. The lowest rated item at all three sites was the availability of on-call rooms and lockers. Significant variability was seen across training sites in the clinical teacher’s ability to act as professional role models, the opportunity for students to apply their previous knowledge to patient care and to independently assess patients before discussion with teachers.

**Conclusion:**

This study tool highlights variation in clinical teaching and supervision at four clinical teaching sites. It provides specific, actionable information which can be utilized to deliver equitable learning experiences across clinical clerkships and teaching sites. It places emphasis on the fact that lack of physical facilities hampers clinical teaching and supervision, hence, on call rooms, lockers and separate rooms for independent student interaction with patients should be provided at all clinical teaching sites.

## INTRODUCTION

There is wide variability in how clinical supervision and teaching takes place at various teaching sites which may lead to inequitable experiences and impact student achievement.^1^ A number of factors contribute to this variation which include responsibility for patient care, time sensitivity of the context, multiple and conflicting commitments, supervisor’s teaching competence, quality of teaching environment, faculty development opportunities and financial pressures.^2^,^3^

The quality of relationship between the supervisor and trainee is critical for effective supervision.^4^ Clinical supervisors should be clinically competent and possess good pedagogical and interpersonal skills to provide direct guidance on clinical work. They should involve the students in joint problem solving, offer constructive feedback, reassurance and support trainees’ empowerment.^1^,^4^ Clinical teaching may be hampered by a lack of clear expectations, absence or ineffective feedback and inappropriate role modelling.^4^ A collaborative relationship with communication, shared expectations and priorities, planning and team work promotes education and contributes to safe, effective patient care.^5^

Where hospitals and medical schools are owned by the same entity or where payment models and contractual requirements exist, clinical teachers are bound to supervise medical students and can be held accountable for it. However, in many cases medical schools do not employ the clinical teachers and therefore, have no direct control over the hospital where clinical teaching takes place. The hospital pays clinical teachers and expects them to teach and supervise but there is no clearly stated contractual requirement.^6^ These issues can undermine the complementary relationship between medical schools and teaching hospitals^7^ leading to variability of clinical experiences across clinical teaching sites.

A similar situation exists at Medical Colleges across United Arab Emirates (UAE). In addition, at the College of Medicine and Health Sciences (CMHS), UAE University (UAEU), several complaints were received from students regarding the quality of clinical teaching and supervision. Few studies have explored issues and variation in clinical teaching and supervision worldwide and in the UAE. Therefore, further investigation was warranted.

The purpose of this study was to explore the variability of clinical teaching and supervision taking place at various clinical teaching sites affiliated with CMHS, Al Ain, UAE. The objective of this study was to:

1. Ascertain overall perception of satisfaction of undergraduate medical students with their clinical clerkship experience.
2. Compare the perceptions of the students about the areas of strength and weakness in clinical teaching and supervision at the four teaching sites.

## METHODS

This descriptive cross-sectional study was conducted at CMHS, UAEU during 2017. The CMHS is affiliated with the UAE University and offers a six-year undergraduate program consisting of two years each of pre-medical and pre-clinical followed by final two years of clinical clerkships. The CMHS relies on a close working relationship with clinical teaching sites and trusts that clinical teachers adhere to clinical supervision guidelines and policies to provide a suitable learning environment. Under a Memorandum of Understanding with Abu Dhabi Health Services Company-SEHA. CMHS utilizes its hospitals in Al Ain and Abu Dhabi for training of clinical students namely Tawam (TH), Al Ain (AH), Ambulatory Health Care Services (AC) Center and Shaikh Khalifa Medical City (SKMC). Students are sent to these training sites, where Clinical Coordinators ensure completion of specified objectives.

### Data collection

Data was collected from all clinical students belonging to years five and six (n=178) rotating at the four clerkship sites affiliated with CMHS. The survey instrument consisted of demographic questions (3 items), questions specific to clinical clerkship teaching and supervision (33 items) derived from existing clinical teaching evaluation instruments^8^-^10^ and one question pertaining to overall evaluation of the clerkship experience. Participants responded using a five point Likert-scale (strongly disagree, disagree, uncertain, agree, strongly agree). Participation in the survey was voluntary, anonymous and informed consent was obtained. A cover letter, consent form and link to web based questionnaire was sent to students by email. Two reminders were sent to maximize response rate. This study was approved by the Ethics Committee (ERS_2017_5575).

### Data Analysis

Demographics were described using descriptive statistics and frequencies. Data was compared by subscales and teaching sites. An individual raw mean score was calculated for each item.^11^ The mean score for each item can vary between 1-5. A mean score of two or less indicates negative perception of the attribute and problems, scores between two and three indicate an area which can be improved while a score above four represents a positive perception of the attribute.^11^ An Exploratory Factor Analysis revealed four subscales. A mean score was also calculated for the subscales^11^,^12^ by teaching sites. The subscale scores reported are mean scores instead of sum scores as this makes comparison of scores between subscales easier.^13^ Analysis of Variance (ANOVA) was used to compare mean score over teaching sites. Data was analyzed using Stata/IC 15 (Stata Corporation, Inc. College Station, TX, UAS). P-values of <0.05 were considered statistically significant.

## RESULTS

A total of 150 clinical students completed the questionnaire, with an overall response rate of 84.4%. The distribution of the participating students by teaching sites, clerkships and years is presented in Table-I. Around 45% and 55% of the students were in clinical year I and II, respectively.

**Table-I:** Distribution of clinical students by clinical teaching sites, clinical year and clinical clerkships.

	Number (%)
Teaching Hospital/Site*	
AH	63 (42.0)
TH	58 (38.7)
AC	15 (10.0)
SKMC	14 (9.3)
Total	150
*Clinical Year-1 Clerkships*	
Internal Medicine-I	21 (31.3)
Surgery-I	12 (17.9)
Pediatrics-I	09 (13.4)
Obstetrics & Gynecology	16 (23.9)
Psychiatry	05 (7.5)
Public Health	04 (6.0)
Total	67
*Clinical Year-11 Clerkships*	
Internal Medicine-II	26 (31.3)
Internal Medicine Selective	02 (2.4)
General Surgery	05 (6.0)
Surgery Specialty	06 (7.2)
Pediatrics-II	10 (12.0)
Family Medicine	15 (18.1)
Emergency Medicine	19 (22.9)
Total	83

* Tawam Hospital (TH), Al Ain Hospital (AH), Ambulatory Health Care Services (AC) Center and Shaikh Khalifa Medical City (SKMC).

Subscale mean score analysis by teaching sites is presented in Table-II. SKMC was perceived as the best by students in terms of teaching and supervision, involvement in direct patient care, patient numbers, clinical findings and availability of physical facilities.

**Table-II:** Subscale mean scores and standard deviation by teaching sites.

	Mean (SD) Scores (max. score 5)				
Subscales	AH	TH	AC	SKMC	p-value
Teacher Characteristics (17items)	3.50 (1.08)	3.84 (0.93)	3.86 (1.21)	4.48 (0.46)	0.008
Involvement in direct patient care (6 items)	3.26 (1.06)	3.85 (1.06)	3.68 (1.19)	4.12 (0.51)	0.03
Patients (4 items)	3.44 (1.02)	3.92 (0.99)	3.70 (1.24)	4.18 (0.64)	0.142
Physical Facilities (5 items)	3.20 (1.09)	3.51 (1.05)	3.05 (1.04)	4.37 (0.47)	0.015

* Tawam Hospital (TH), Al Ain Hospital (AH), Ambulatory Health Care Services (AC) Center and Shaikh Khalifa Medical City (SKMC).

The distribution of the items by subscales and mean ranges is shown in Table-III. The range of mean rating was between 2.33±1.23-4.79±0.43. SKMC was rated as the best clinical teaching site with mean rating of 3.79±0.97-4.79±0.43. The highest rated item was clinical teacher’s promotion of critical thinking in medical students while the lowest rated item was the opportunity to take responsibility for patient care. AC site had a mean rating of 2.33±1.23-4.13±1.19. The highest rated item at this site was the clinical teacher encouraging students to ask questions and participate actively. At TH, the mean ratings ranged between 2.65±1.64-4.31±0.86 with highest rated item being ability of the students to see cases with positive clinical findings. At the Al-Ain Hospital, the mean rating was in the range of 2.79±1.45-3.81±1.11. The item rated highest here was the ability of students to see cases with positive clinical findings. The lowest rated item at all three sites was the availability of on-call rooms and lockers.

**Table III:** Mean Scores and standard deviation of individual items by subscales and teaching sites.

		Mean (SD) Scores (max. score 5)				
Subscale/Item No/Items of CCSTQ		AH	TH	AC	SKMC	p-value

**Subscale 1-Teacher Characteristics**						
6	I have the space and opportunity to reflect on and prepare report after the clinical encounter.	3.16 (1.33	3.59 (1.2)	3.4 (1.59)	4.57 (0.51)	0.003
18	It was easy for me to access the hospital teacher.	3.19 (1.29)	3.6 (1.18)	3.8 (1.26)	3.93 (1)	0.08
19	My hospital clinical teacher fosters an environment of respect in which I feel comfortable participating.	3.48 (1.18)	3.86(1.03)	3.73(1.33)	4.57 (0.51)	0.009
20	My hospital clinical teacher is enthusiastic and committed to teaching.	3.48 (1.32)	3.76(1.19)	3.8 (1.37)	4.71 (0.47)	0.01
21	My hospital clinical teacher is available.	3.47 (1.28)	3.81(1.13)	3.87(1.36)	4.64 (0.5)	0.009
22	My hospital clinical teacher is punctual.	3.69 (1.15	3.84(1.17	3.67(1.29	4.57 (0.51	0.069
23	My hospital clinical teacher shows genuine concern for my learning.	3.45 (1.21)	3.71(1.18)	3.67(1.29)	4.71 (0.61)	0.005
24	My hospital clinical teacher has reasonable expectations of me.	3.61 (1.12)	3.91 (1.1)	3.6 (1.18)	4.29 (0.61)	0.126
25	My hospital clinical teacher has good communication skills.	3.71 (1.22)	4.1 (0.85)	4 (1.31)	4.5 (0.52)	0.043
26	My hospital clinical teacher gives me opportunity to offer my opinion on patient problems / management.	3.53 (1.25)	3.83(1.19)	4 (1.13)	4.64 (0.5)	0.013
27	My hospital clinical teacher encourages me to think.	3.73 (1.16)	4.05 (1)	4.07(1.16)	4.79***(0.43)	0.008
28	My hospital clinical teacher asks me questions (clarifying, probing, reflective) that stimulate learning.	3.71 (1.18)	3.9 (0.97)	4.07 (1.16)	4.57 (0.51)	0.048
29	My hospital clinical teacher encourages me to ask questions and participate actively.	3.58 (1.21)	3.79(1.18)	4.13***(1.19)	4.43 (0.51)	0.057
30	My hospital clinical teacher is a professional role model.	3.48 (1.24)	4.05(0.94)	4 (1.31)	4.71 (0.61)	<0.001
31	I am given enough assignments during my clinical rotation.	3.61 (1.27)	4.05(1.03)	4 (1.31)	4.21 (0.8)	0.12
32	My hospital clinical teacher offers timely, constructive feedback.	3.35 (1.24)	3.72(1.23)	3.87 (1.13)	4.14 (1.1)	0.09
33	My hospital clinical teacher offers suggestions for my development.	3.32 (1.33)	3.67(1.29)	3.93 (1.1)	4.14 (1.03)	0.085

**Subscale 2–Involvement in direct patient care**						

8	The time spent with patients is adequate for my clinical learning.	3.27 (1.3)	3.66(1.36)	3.73(1.28)	4.14 (0.77)	0.092
13	I am given the opportunity to have first-hand contact experience with patients.	3.34 (1.19)	3.81(1.21)	3.4 (1.24)	3.93 (0.83)	0.096
14	I am actively involved in the patient care.	3.13 (1.34)	3.66(1.42)	3.4 (1.18)	4.29 (0.61)	0.015
15	I have opportunity to apply my previous knowledge to patient care.	3.31 (1.26)	4.1 (1.02)	3.73(1.22)	4.36 (0.63)	<0.001
16	I have the opportunity to take responsibility for patient care.	2.95 (1.21)	3.62(1.37)	3.67(1.35)	3.79**(0.97)	0.012
17	I have the opportunity to communicate with patients and their families.	3.58 (1.19)	4.24(0.88)	4.13(1.41)	4.21 (0.8)	0.006

**Subscale 3–Patients**						

9	I have seen a sufficient number of clinical cases.	3.21 (1.23)	3.86(1.28)	3.87 (1.3)	4.14 (0.77)	0.008
10	I have seen a sufficient variety of clinical cases.	3.24 (1.17)	3.71(1.32)	3.93(1.28)	3.86 (1.03)	0.072
11	I have seen cases with positive clinical findings.	3.81***(1.11)	4.31***(0.86)	4.0 (1.31)	4.57 (0.51)	0.013
12	I have seen some unusual/rare clinical cases.	3.5 (1.2)	3.81(1.23)	3.0 (1.56)	4.14 (0.77)	0.042

**Subscale 4–Physical Facilities**						

1	I have suitable space, computer/internet access and access to relevant patient information.	3.61 (1.38)	3.72 (1.36)	2.93 (1.53)	4.43 (0.51)	0.029
2	There are adequate numbers of on-call rooms and lockers for clinical students and interns.	2.79** (1.45)	2.65** (1.64)	2.33** (1.23)	4.21 (1.31)	0.003
3	There is adequate transport facility for my transportation to and from hospital.	3.4 (1.42)	3.98 (1.01)	3.4 (1.12)	4.0 (1.04)	0.042
4	I have room to independently assess patients before discussion with the hospital clinical teacher.	3.0 (1.31)	3.57 (1.42)	3.27 (1.58)	4.64 (0.5)	<0.001
5	I have the space and opportunity to prepare before the clinical encounter.	3.19 (1.27)	3.67(1.25)	3.33(1.35)	4.57 (0.51)	0.002

* Tawam Hospital (TH), Al Ain Hospital (AH), Ambulatory Health Care Services (AC) Center and Shaikh Khalifa Medical City (SKMC).

** lowest mean rating,

*** highest mean rating.

## DISCUSSIONS

Overall student perceptions about their clinical clerkship experience was positive. With SKMC being perceived as best teaching site in terms of clinical teaching and supervision. Analysis of data by subscales and teaching sites revealed areas of strength and those needing improvement. Significant variation was observed across training sites in the clinical teacher’s ability to act as professional role models, the opportunity for students to apply their previous knowledge to patient care and to independently assess patients before discussion with teachers.

The low rating accorded to the availability of on-call rooms and lockers at three of the four sites highlights an important deficiency. International bodies have been unanimous in emphasizing availability of physical facilities for clinical teaching. According to LCME accreditation guidelines^14^ stipulation 5.11:

“A medical school ensures that its medical students have, at each campus and affiliated clinical site, adequate study space, lounge areas, personal lockers or other secure storage facilities, and secure call rooms…….”

Similarly, in setting standards and requirements for delivery of medical education and training, the General Medical Council specifies under the theme of learning environment and culture that it is the responsibility of local education providers to make available the facilities needed to deliver the clinical curriculum.^15^

Students’ perception of a lack of supportive clinical learning environment, may contribute to anxiety associated with a sense of external obstacles to personal accomplishment and effectiveness.^16^,^17^ This finding indicates an important challenge of balancing enhancement of both patient and student-centered environments and a deficiency at three training sites. Another related deficiency emphasized at three of the four training sites was the absence of rooms to independently clerk patients before discussion with the attending. Planning is important for optimal teaching in outpatient clinics. DaRosa and colleagues reported “wave scheduling” of patient appointments.^18^ Dent identified other strategies such as breakout, supervising and report back.^19^ All these time efficient strategies require rooms for students to interact independently with patients.

Another area requiring attention at all four sites is the opportunity to take responsibility for patient care. This deficiency may partly be due to the nature of practice in our context which is private with priority for increasing patient volume. This may lead to clinicians’ reluctance to actively involve students in patient care, impacting student learning and their perception of not being part of the team. Some strategic planning and minor changes can motivate students to feel part of the `community of practice’.^20^ Several models have been described in literature such as the One Minute Preceptor (OMP) Model^21^, the SNAPPS model^22^, the Aunt Minnie Model^23^ and the Activated Demonstration Model^24^ for teaching skills. All these models improve the effectiveness of outpatient teaching and maximize utilization of time whilst involving students in patient care.

The strength of the teacher–learner relationship impacts the quality of teaching.^25^ This relationship has multiple facets including access to the clinical teacher. Access to the teacher was rated low at all four teaching sites. Whilst it is too much to expect that the clinical teachers will be available at all times, the expectation is that some convenient time will be allocated.

The large standard deviation for many items indicates considerable variation from clinician to clinician which reflects a reality of clerkship teaching and learning. This emphasizes the need for faculty development and standardization not only across teaching sites but also within to contain variation within acceptable limits.

The high response rate is a strength of the study. This study was conducted at a single medical school but the multiyear and multisite approach of the current study, contributes to the representativeness of the study population. The findings are generalizable to other contexts where clerkship teaching and supervision in similar situations and faces similar constraints and challenges especially when the teaching hospital is not owned by the medical school.

### Limitations of the study

It includes the exclusive focus on students’ perspective. Further work is required to explore perceptions of clinical teachers and coordinators. Another limitation was that owing to the small sample size, data was not analyzed for differences across clerkships.

## CONCLUSIONS

This study highlights variation in clinical teaching and supervision at clinical teaching sites. It provides specific, actionable information which can be utilized to deliver equitable learning experiences across clinical clerkships and teaching sites. Stake holders such as clinical clerkship coordinators, clerkship directors at the medical schools and academic affair directors in hospitals can institute a shared, quality improvement plan to achieve equitable learning experiences for clerkship students across clerkships and across clinical teaching sites. The study emphasizes that clinical teaching and supervision is much more complex than often acknowledged. The interrelated supportive nature of quality human and physical resources deserves further attention. A lack of physical facilities hampers clinical teaching and supervision, hence, on call rooms, lockers and separate rooms for independent student interaction with patients should be provided at all clinical teaching sites.

### Authors’ Contribution

**NN:** Contributed to the conception, acquisition, analysis, and interpretation of data, drafting and revising the manuscript.

**ME:** Contributed to revising manuscript critically for important intellectual content.

**MA:** Contributed to conception of the work and collection of data.

**LAA:** Contributed to analysis and interpretation of data.

All authors take responsibility and approve the final manuscript.
